# Standardization of Laparoscopic Pelvic Examination: A Proposal of a Novel System

**DOI:** 10.1155/2013/153235

**Published:** 2013-12-30

**Authors:** Mohamed A. Bedaiwy, Rachel Pope, Drisana Henry, Kristin Zanotti, Sangeeta Mahajan, William Hurd, Tommaso Falcone, James Liu

**Affiliations:** ^1^Department of Gynecology, University Hospitals Case Medical Center, Case Western Reserve University, Cleveland, OH 44106, USA; ^2^Division of Reproductive Endocrinology and Infertility, Department of OB/GYN, University of British Columbia, D415A, Shaughnessy Building-4500 Oak Street, Vancouver, BC, Canada V6H 3N1; ^3^Department of OB/GYN and Women's Health Institute, Cleveland Clinic, Cleveland, OH 44106, USA

## Abstract

*Objective*. Laparoscopic pelvic assessment is often performed in a nonstandardized fashion depending on the surgeon's discretion. Reporting anatomic findings is inconsistent and lesions in atypical locations may be missed. We propose a method for systematic pelvic assessment based on anatomical landmarks. *Design*. Retrospective analysis. *Setting*. Tertiary care academic medical center. *Intervention*. We applied this system to operative reports of 540 patients who underwent diagnostic or operative laparoscopy for unexplained infertility between 2006 and 2012. The pelvis was divided into 2 midline zones (zone I and II) and right and left lateral zones (zone III and IV). All reports were evaluated for the comprehensiveness of description with respect to normal findings or pathology for each zone. *Results*. Of 540 surgeries, all reports commented on the uterus, tubes, and ovaries (100%), but only 17% (*n* = 93, 95% CI: 13.8–20.2) commented on the dome of the bladder and the anterior cul-de-sac. 24% (*n* = 130, 95% CI: 20.4–27.6) commented on the posterior cul-de-sac, and 5% (*n* = 29, 95% CI: 3.2–6.8) commented on the pelvic sidewall. Overall, 6% (*n* = 34, 95% CI: 4–8) reported near complete documentation of the pelvic zones. *Conclusion*. Implementation of a systematic approach for laparoscopic pelvic examination will enhance the diagnostic accuracy and provide better communication between care providers. In the absence of pelvic pathology, we recommend a minimum of 6 photographs of the 6 pelvic zones.

## 1. Introduction

Years after surgical procedures are performed, operative reports are often the only source of information another surgeon possesses when attempting to understand the history and internal anatomy of a patient. Evidence shows that a structured format for documenting findings improves overall accuracy of reporting and, by extension, is likely to improve patient outcomes [1, 2]. An appropriately detailed report may greatly improve treatment strategy and general preparedness for a case, theoretically leading to better patient safety and care. While efforts have been made in the general surgical field to improve and standardize operative reports, these efforts are still lacking in gynecology surgery.

Pelvic anatomy is unique in that various pathologies can be missed if not intentionally sought out for identification. These anatomical characteristics could influence the detailed description of pelvic findings during surgery in general and, more specifically, during laparoscopy. Classically the pelvis is divided into a true and false pelvis. While the false pelvis is the space enclosed by the pelvic girdle above and in front of the pelvic brim and considered part of the abdominal cavity, the true pelvis includes the genital tract midline between the lower end of the urinary tract anteriorly and the gastrointestinal tract posteriorly. The ligamentary attachments of the female genital organs add to the anatomical uniqueness of the pelvis. For instance, the round ligament, which extends from the cornua to the internal ring, could harbor pathology from its origin to its insertion. The uterosacral ligaments and the suspensory ligaments of the ovary are often inspected but not described. Other anatomically obscure locations include the ovarian fossa, the lateral pelvic sidewall, and the area inferior to the uterosacral ligament.

The objective of this study is to propose a method for systematic pelvic assessment based on anatomical landmarks and structured documentation with laparoscopic photography. To illustrate the current deficiencies, we retrospectively applied this system to a cohort of patients who underwent laparoscopy for unexplained infertility to assess the comprehensiveness of the operative reports.

## 2. Materials and Methods

### 2.1. Proposed System

**Figure 1:**
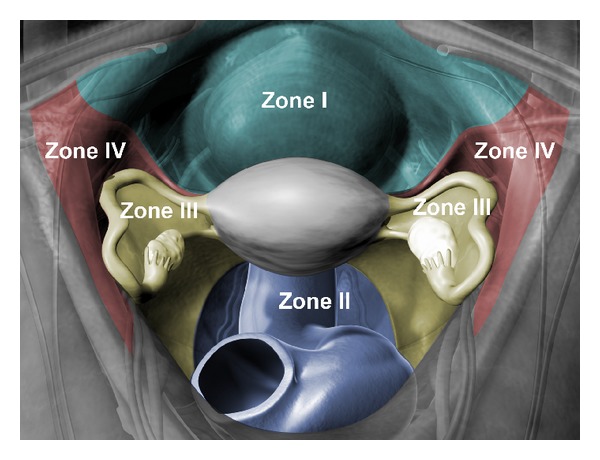
A color-coded illustration of the anatomical boundaries and the contents of all pelvic zones.

In our proposed system, the pelvis was topographically divided into two midline zones (zone I & II) and two paired (right and left) lateral zones (zone III & IV). Zone I is the area between the two round ligaments from their origin at the uterine cornua to their insertion in the deep inguinal rings. Zone II is the area between the two uterosacral ligaments from their origin from the back of the uterus to their insertions in the sacrum posteriorly. Zone III is the area between the uterosacral ligament inferiorly and the entire length of the fallopian tube and the infundibulopelvic ligament superiorly. Zone IV is the triangular area lateral to the fallopian tube and the infundibulopelvic ligament and medial to the external iliac vessels up to the round ligament (Figure 1). The contents of the different zones are shown in Table 1.

### 2.2. Retrospective Evaluation of Dictated Reports

This study was conducted at the University Hospitals Case Medical Center (UHCMC), Case Western Reserve University, Cleveland, Oh, USA. After IRB approval was obtained, operative reports of 540 patients who underwent diagnostic or operative laparoscopy for the diagnosis of unexplained infertility between January 2005 and January 2012 were collected. The operative reports for these patients were reviewed with allocation of the reported positive or negative findings to the respective zones as shown above. All reports were evaluated for the comprehensiveness of the description with respect to normal findings or pathology for six zones as follows. Using this mapping of the pelvis, the operative reports were reviewed for completeness in description of anatomical findings. Descriptive statistics are presented.

## 3. Results

During the review period of the study, 8876 laparoscopies and hysteroscopies were performed within the entire UHCMC system for a variety of indications. Of these, a total of 540 cases of diagnostic and/or operative laparoscopy with and without hysteroscopy for unexplained infertility were identified. These cases were selected as they are usually intended as a careful surveillance of pelvic anatomy in order to identify an etiology of infertility. As the goal of these surgical cases is the identification of anatomy, it was thought fit that these operative reports would focus on the description of anatomy. All operative reports commented on the uterus, tubes, and ovaries (100%), which reflect parts of zone I and part of zone III. Only 17% (*n* = 93, 95% CI: 13.8–20.2) commented on the dome of the bladder and the anterior cul-de-sac (the remainder of zone I). Twenty-four percent (*n* = 130, 95% CI: 20.4–27.6) commented on the posterior cul-de-sac, which represents part of zone II. Interestingly, only one fourth of those who addressed zone II (6%; *n* = 34, 95% CI: 4–8) commented on the rectosigmoid. Moreover, only 5% (29/540) commented on the pelvic sidewall peritoneum without specifying whether the ovarian fossa and the peritoneum overlying zone IV were evaluated. Overall, only 6% (*n* = 34, 95% CI: 4–8) reported either positive and/or negative findings in the various pelvic zones resulting in complete documentation of the presence or absence of pelvic findings (Table 2). Supplemental photographic documentation of all pelvic areas was frequently missed; it was found only in 6% (*n* = 34, 95% CI: 4–8) of patients' charts.

## 4. Conclusion

The paucity of detail in operative reporting represents a missed opportunity to document important anatomical findings that could prove useful in future patient care. Our retrospective chart review demonstrated that description of important pelvic structures is frequently missing in operative notes from diagnostic and operative laparoscopy. The anterior cul-de-sac, deep inguinal rings, ovarian fossa, and the lateral pelvic sidewall peritoneum are the most frequently missed areas. Photographic documentation of normal and abnormal findings was also frequently missed.

As seen in the general surgical literature, standardizing operative reporting improves completeness of documentation [2]. If such systems are in place, residents can be taught these methods for reporting during their training [3, 4]. As the era of digital photography and electronic medical records evolves, this is a very appropriate time to innovate with respect to the methods by which we document our surgical findings. Implementation of a systematic approach for laparoscopic pelvic examination will indeed enhance the diagnostic accuracy, help diagnose lesions in anatomically challenging locations, and provide the required standardization with its clinical and academic advantages. Templates have been created to achieve standardization in general operative reports [5]. Photographic documentation of these anatomic regions would provide an additional advantage.

We recommend a minimum of 6 photographs of the 6 pelvic zones in the absence of pelvic pathology. These six zones are depicted in Figure 1. Images of these zones will supplement the report. In addition, if surgeons dictate according to the zones, comprehensive details will be incorporated into the description report. Two copies of photos should be available for charting.

In summary, a comprehensive description of important pelvic structures is frequently missing in operative notes from diagnostic and operative laparoscopy. The anterior cul-de-sac, deep inguinal rings, and the lateral pelvic sidewall peritoneum are the most frequently missed areas. A large proportion of gynecological surgery utilizes operative and diagnostic laparoscopy. Intraoperative photographic documentation is a true benefit to this approach. Lack of a standardized protocol for photographic documentation is a missed opportunity in providing quality patient care.

The advantages of our proposed system are several. First, it is based on anatomical landmarks, which allow standardization. Second, it is comprehensive as it includes all pelvic major structures such as the bladder, uterus, adnexa, and the rectosigmoid colon. It also covers supportive pelvic structures such as the round ligaments, the broad ligament, and the uterosacral ligament. Moreover, it describes peritoneal surfaces such as the anterior and the posterior cul-de-sac and the ovarian fossa. In addition, it covers frequently missed areas such as the internal rings and the triangular peritoneal area lateral to the fallopian tube and the infundibulopelvic ligament. Lastly, it is easy to follow system whereas the examination could be performed in anteroposterior, then lateral fashion where zone I and II will be examined first (midline zones). Subsequently, lateral zones (right zones III and IV followed by left zones III and IV) are to follow. Alternatively, clockwise or counterclockwise examination could be performed.

The main limitation of this study was the retrospective use of sources to validate the use of our novel system. In the future, we plan to use operative reports that include photography in order to prospectively describe the six pelvic zones in order to validate this method. By doing this, we propose that more pathology will be diagnosed resulting in improved patient care and communication between surgeons will be improved by extension.

## Figures and Tables

**Table 1:** Descriptive summary of the anatomical boundaries and the contents of each pelvic zone.

Zone	Boundaries	Contents
Zone I	Midline anterior abdominal cavity limited by the round ligaments bilaterally	(1) Uterine dome and anterior surface (2) Anterior surface of the broad ligaments (3) Bladder dome (4) Internal ring and inferior epigastric vessels

Zone II	Midline posterior zone of the abdominal cavity limited by the uterosacral ligaments bilaterally	(1) Uterine dome and posterior surface (2) Pouch of Douglas (3) Rectovaginal septum (4) Sigmoid colon (5) Presacral peritoneum

Zone III	Lateral pelvic sidewalls limited by the uterosacral ligament and the adnexae and infundibulopelvic ligaments	(1) Fallopian tube and ovary (2) Posterior surface of the broad ligament (3) Ovarian fossa (4) Vessels and ureter

Zone IV	Pelvic sidewall limited by the round ligament, adnexae and infundibular ligament, and external iliac vessels	(1) External iliac vessels (2) Ilioinguinal nerve

**Table 2:** Percentages of the surgical reports that described findings in any structure or all structures of every pelvic zone.

Zone	Percentage and/or (95% CI) of reports that included any part of this zone	*n*	Percentage and/or (95% CI) of reports that included all aspects of this zone	*n*
I	100%	540	17% (13.8–20.2)	93
II	24% (20.4–27.6)	130	6% (4–8)	34
III	100%	540	0%	0
IV	5% (2–6.8)	29	0%	0
